# Polyamine metabolism related gene index prediction of prognosis and immunotherapy response in breast cancer

**DOI:** 10.3389/fonc.2025.1613458

**Published:** 2025-07-31

**Authors:** Ruoya Wang, Shouliang Cai, Qing Gao, Yidong Chen, Xue Han, Fangjian Shang, Chunyan Liang, Guolian Zhu, Bo Chen

**Affiliations:** ^1^ Department of Otolaryngology, The First Affiliated Hospital of Jinzhou Medical University, Jinzhou, China; ^2^ Department of Thyroid and Breast Surgery, Ansteel General Hospital, Anshan, China; ^3^ Department of Breast and Thyroid Surgery, Linyi Maternal and Child Healthcare Hospital, Linyi, China; ^4^ Department of Breast Surgery, The First Hospital of China Medical University, Shenyang, China; ^5^ Breast Thyroid Surgery Ward 4, Affiliated Zhongshan Hospital Of Dalian University, Dalian, Liaoning, China; ^6^ Department of General Surgery, the Fourth Affiliated Hospital of China Medical University, Shenyang, Liaoning, China; ^7^ The First Department of Oncology, The Fourth Hospital of China Medical University, Shenyang, China; ^8^ Department of Breast Surgery, The Fifth People’s Hospital of Shenyang, Shenyang, Liaoning, China

**Keywords:** polyamine metabolism-related genes, breast cancer, multi-omics, METABRIC, prognostic, tumor microenvironment

## Abstract

**Background:**

Polyamine metabolism is closely associated with tumorigenesis, progression, and the tumor microenvironment (TME). This study aimed to determine whether polyamine metabolism-related genes (PMRGs) could predict prognosis and immunotherapy efficacy in Breast Cancer (BC).

**Methods:**

We conducted a comprehensive multi-omics analysis of PMRG expression profiles in BC. Consensus cluster analysis was used to identify PMRG expression subtypes in the METABRIC cohort. Univariate and multivariate Cox regression analyses were performed to identify independent prognostic genes, which were subsequently used to construct a predictive model for BC, along with a novel nomogram based on PMRGs. The model was validated using an independent cohort (GSE86166). Independent prognostic genes were further verified in BC tissues using quantitative real-time PCR (qRT-PCR), Semi-quantitative Western blot, and immunohistochemistry. Additionally, we analyzed the immune microenvironment and enriched pathways across different subtypes using multiple algorithms. Finally, the “oncoPredict” R package was used to assess potential drug sensitivities in high-risk and low-risk groups.

**Results:**

Seventeen polyamine metabolism genes were identified. PMRGs were abundantly expressed in tumor cells, with 12 survival-related genes being selected. In the METABRIC cohort, two PMRG expression subtypes were identified, with cancer- and immune-related pathways being more active in cluster B, which was associated with a worse prognosis. Six genes were used to construct a prognostic model through univariate and multivariate Cox regression analyses. The predictive performance of the polyamine metabolism model was validated by ROC curve analysis (training cohort: METABRIC, AUC3years=0.684; validation cohort: GSE86166, AUC3years=0.682). A nomogram combining risk scores and clinicopathological features was constructed. Decision Curve Analysis (DCA) demonstrated that the model could guide clinical treatment strategies. Four high-risk independent prognostic factors (*OAZ1*, *SRM*, *SMOX*, and *SMS*) were validated as being upregulated in breast cancer tissues. The model successfully stratified BC patients into high-risk and low-risk groups, with the high-risk group exhibiting poorer clinical outcomes. Functional analysis revealed significant differences in immune status and drug sensitivity between high-risk and low-risk groups.

**Conclusions:**

This study elucidated the biological characteristics of PMRG expression subtypes in BC, identifying a polyamine-related prognostic signature and four novel biomarkers to accurately predict prognosis and immunotherapy response in BC patients.

## 1 Introduction

Polyamines (PAs) are small polycationic alkyl amines, including putrescine, spermidine, and spermine. Polyamines fulfill important cellular functions not only in eukaryotes, but in virtually all organisms, including prokaryotes as well (1). Polyamines exist in mammalian cells at millimolar concentrations. These molecules contain multiple amino groups, primarily produced by the decarboxylation of specific amino acids, and are essential for normal cell growth and development in eukaryotic organisms. PAs are involved in various cellular activities through interactions with negatively charged DNA, RNA, or proteins (2, 3), and their depletion leads to cell stagnation. They participate in stabilizing cell structures, binding nucleic acids, and biosynthesizing proteins (4, 5). Mutations in polyamine metabolism (PM) enzymes or transporters are linked to Snyder-Robinson syndrome and other diseases (6–9).

Breast cancer is the second most common cancer worldwide, affecting approximately 42,000 women annually and representing the leading cause of cancer-related deaths among females (10). Due to the high heterogeneity of BC treatments are increasingly targeted toward subtypes, stages, and grades (11). Conventional therapies for BC include surgery, chemotherapy, and radiotherapy (12). Currently, immunotherapy has gained significant attention, encompassing immune checkpoint blockade, vaccines, immune-oncolytic virus drugs, and adoptive cell therapy (13). The advancement of targeted and immunotherapy approaches has expanded treatment options, particularly for advanced cases. However, many patients fail to respond effectively, highlighting the need to identify novel biomarkers that can accurately predict immunotherapy response.

Many evidence strengthens the hypothesis that a rise in intracellular PA concentrations, mainly through an up-regulation of PA biosynthetic enzymes, is associated with increased cell proliferation and is usually linked to tumorigenesis (14–16). Dysregulated PM has been observed in various cancers, including breast, colorectal, prostate, skin, renal, and lung cancers (17–24). Elevated PA levels have also been detected in body fluids of cancer patients (25, 26). PM functions downstream of major oncogenic pathways (27), and inhibitors of key biosynthetic enzymes in PM pathways can significantly impact tumor progression. Thus, PM is closely associated with tumorigenesis and represents a key target for cancer therapy. While targeted PMRG therapies offer a promising approach for BC, it remains unclear how PM affects the tumor microenvironment (TME) and its role in immunotherapy.

In this study, we identified 17 PMRGs from the literature and combined multi-omics data from the METABRIC, TCGA, and GEO datasets, including transcriptomics, single-cell sequencing, and copy number variation analyses. We classified two PMRG expression subtypes in the METABRIC cohort, uncovering significant differences in immune cell infiltration and pathways, suggesting that PM strongly influences TME characteristics. Using univariate and multivariate Cox analyses, we identified six prognostic genes to develop a risk score model for predicting patient survival, which was validated in the independent GSE86166 cohort. Furthermore, we confirmed the expression of key prognostic genes (*OAZ1*, *SRM*, *SMOX*, *SMS*) in clinical tissues through qRT-PCR, Western blot, and immunohistochemistry assays. The PMRG model revealed distinct immune features and immunotherapy responses across BC subtypes, enabling the prediction of clinical responses to chemotherapy. This is the first study to comprehensively investigate the role of PMRGs in BC patients from the perspectives of prognosis and immunotherapy response.

## 2 Materials and methods

### 2.1 Data Collection

The RNA sequencing expression profile data for BC samples were collected from the TCGA database (https://portal.gdc.cancer.gov/), including 113 normal and 1113 cancer cases. The training dataset was obtained from the Molecular Taxonomy of Breast Cancer International Consortium (METABRIC) database, comprising 1,992 cases (https://www.cbioportal.org/). The validation dataset (GSE86166) included 305 cases from the Gene Expression Omnibus (GEO) database (http://www.ncbi.nlm.nih.gov/geo/). The single-cell dataset GSE161529 was sourced from the Tumor Immune Single-Cell Hub (TISCH; http://tisch.comp-genomics.org) (28).

### 2.2 Expression of genes related to polyamine metabolism and protein-protein interaction network

We collected 17 genes related to polyamine metabolism from the literature (29) (**Supplementary Table S1**) and compared their expression levels in tumor tissues and adjacent normal tissues using the TCGA database. A protein-protein interaction (PPI) network was constructed using the STRING platform (https://string-db.org/) to analyze the interconnections among these genes (30).

### 2.3 Copy number variation analysis

Copy number variation (CNV) is common in cancers and often serves as a driving event. Chromosomal regions are frequently lost or gained in cancer patients, and CNVs are significantly associated with BC risk (31). Breast cancer CNV data were downloaded from XENA (https://xena.ucsc.edu/) to investigate alterations in polyamine metabolism genes and their chromosomal locations.

### 2.4 Consensus clustering

Consensus cluster analysis was performed using the “ConsensusClusterPlus” R package. The K-means method was employed to identify distinct patterns related to polyamine metabolism gene expression. Principal component analysis (PCA) was conducted to analyze clustering of the two subtypes, and the “ggplot2” R package was used to validate clustering reliability with the uniform manifold approximation and projection (UMAP) method.

### 2.5 Functional enrichment analysis

We downloaded “c2.cp.kegg.symbols.gmt” and “c5.go.symbols.gmt” data from the MSigDB database (https://www.gsea-msigdb.org/gsea/msigdb) and performed gene set variation analysis (GSVA) using the “GSVA” R package (32).

### 2.6 Development and validation of prognostic features based on polyamine metabolism-related genes

Univariate Cox regression analysis was conducted on the training METABRIC cohort to identify survival-associated genes. A multivariate Cox regression model was subsequently applied to determine key genes and calculate their coefficients for both the training cohort (METABRIC) and the validation cohort (GSE86166). Six polyamine metabolism-related genes closely associated with overall survival (OS) were identified. The risk score was calculated as follows:


$$\begin{array}{c} \text{Risk}\;\text{score}=\left(-0.2114\;*\;ATP13A2\right)+\left(0.53550\;*\;OAZ1\right)+\\ \left(-0.39230\;*\;PAOX\right)+\left(0.21820\;*\;SMOX\right)\\ +\left(0.41053\;*\;SRM\right)+\left(0.1241\;*\;\text{SMS}\right). \end{array}$$

The model’s predictive performance was assessed using Kaplan-Meier (KM) survival curves and receiver operating characteristic (ROC) curve analysis over time.

### 2.7 Construction and evaluation of prediction Nomogram

The “survival” R package was used to compare the prognosis of high-risk and low-risk groups in the METABRIC and GSE86166 BC cohorts. A prognostic nomogram based on the six independent prognostic genes was developed using the “rms” R package. This nomogram predicted 1-, 3-, and 5-year survival rates for BC patients. DCA was performed to evaluate the clinical net benefit (33).

### 2.8 Relationship between risk score and immune cell infiltration

CIBERSORT and ssGSEA R scripts were used to quantify risk scores and immune cell infiltration (34). CIBERSORT was applied to estimate the proportions of immune cell types in high-risk and low-risk groups, with the sum of immune cell type scores equaling 1 for each sample. Spearman correlation analysis was performed to investigate the relationship between risk scores and immune-infiltrating cells.

### 2.9 Immune characteristic analysis

Tumor mutation burden (TMB) for each patient in the METABRIC cohort was evaluated using the “ESTIMATE” software package (35). Differences in TMB between high-risk and low-risk subgroups, including Stromal Score, Immune Score, and ESTIMATE Score, were analyzed.

### 2.10 Chemotherapy response prediction

The “oncoPredict” package was used to predict the sensitivity of therapeutic agents for the different subgroups (36).

### 2.11 Patient samples

Human BC specimens and nearby nontumorigenic specimens were collected from 40 diagnosed BC patients without preoperative treatment during surgery from June 2018 to October 2022 at the First Affiliated Hospital of China Medical University. Among them, 20 samples were embedded in paraffin for immunohistochemical analysis; The remaining samples were all rapidly frozen in liquid nitrogen and then stored at -80°C for future use. This study was approved by the Ethics Committee of the First Affiliated Hospital of China Medical University.

### 2.12 qRT-PCR

Fifteen paired cancer and adjacent tissue samples from BC patients were collected at our institution and stored at -80°C. Total RNA was extracted using Trizol (Sigma) following the manufacturer’s protocol, and RNA quality was assessed using a Nanodrop (Thermo). cDNA was synthesized via reverse transcription using Hiscript QRT supermix for qPCR (Vazyme). QRT-PCR was performed using SYBR Green Mastermix (Vazyme), with β-actin serving as the reference gene. Expression levels were quantified using the 2-ΔΔCt method. Primer sequences for target genes are listed in **Supplementary Table S2**.

### 2.13 Western blot

Five paired cancer and adjacent tissue samples from BRCA patients were collected and stored at -80°C. Protein concentration was measured using Beyotime reagents (China). Twenty micrograms of protein were mixed with 5× SDS loading buffer, separated via 10% sodium dodecyl sulfate-polyacrylamide gel electrophoresis (SDS-PAGE), and transferred to a PVDF membrane (Millipore). The membrane was blocked with 3% BSA buffer in PBS at room temperature for 1 hour, followed by overnight incubation at 4°C with primary antibodies diluted in 3% BSA buffer. The next day, membranes were washed with TBST and incubated with specific secondary antibodies at room temperature for 2 hours. Protein expression 33was detected using an ECL chemiluminescence kit (Beyotime).

Antibodies used in this study:

- Anti-*OAZ1* (Ornithine Decarboxylase Antizyme 1; 1:1000; Rabbit; cat. no. ab223481; Abcam).
- Anti-*SMOX* (Spermine Oxidase; 1:1000; Rabbit; cat. no. ab213631; Abcam).
- Anti-*SMS* (Spermine Synthase; 1:1000; Rabbit; cat. no. ab241496; Abcam).
- Anti-*SRM* (Spermidine Synthase; 1:1000; Rabbit; cat. no. ab156879; Abcam).
- Anti-*β-actin* (Beta Actin; 1:3000; Rabbit; cat. no. AF7018; Affinity).
- Goat Anti-Rabbit IgG (H+L) HRP (1:3000; cat. no. S0001; Affinity).

### 2.14 Immunohistochemistry

Paraffin-embedded tumor tissue sections were deparaffinized, rehydrated, and incubated overnight at 4°C with primary antibodies against *OAZ1*, *SMOX*, *SRM*, and *SMS*. The sections were then incubated with the corresponding anti-rabbit/mouse secondary antibodies (Zhongshan, China) at 37°C for 2 hours. Sections were treated with ABC-peroxidase and diaminobenzidine (DAB) (Zhongshan, China), counterstained with hematoxylin, and visualized using light microscopy.

### 2.15 Statistical analysis

The data were analyzed by R software (version 4.3.1, https://www.r-project.org/). R packages (ESTIMATE, ggplot2, GSVA, limma, survminer, and survival) were applied for data analysis and graph plotting. The median value of risk scores was treated as the cutoff value for the two subgroups. The qRT-PCR results were analyzed by GraphPad Prism (version 10.1.2). Student’s T-test was employed to compare the statistical differences between the two groups. The Kaplan– Meier method was performed for prognosis among groups. Multivariate COX analysis was used to screen prognostic related genes. The Pearson test was used for correlation analysis. A *P* value < 0.05 was considered statistically significant (*, p < 0.05; **, p < 0.01; ***, p < 0.001), and the false detection rate (FDR) *q* < 0.05 was used for multiple testing correction.

## 3 Result

### 3.1 Multiomics analysis of polyamine metabolism-related genes in Breast caner

**Figure 1:**
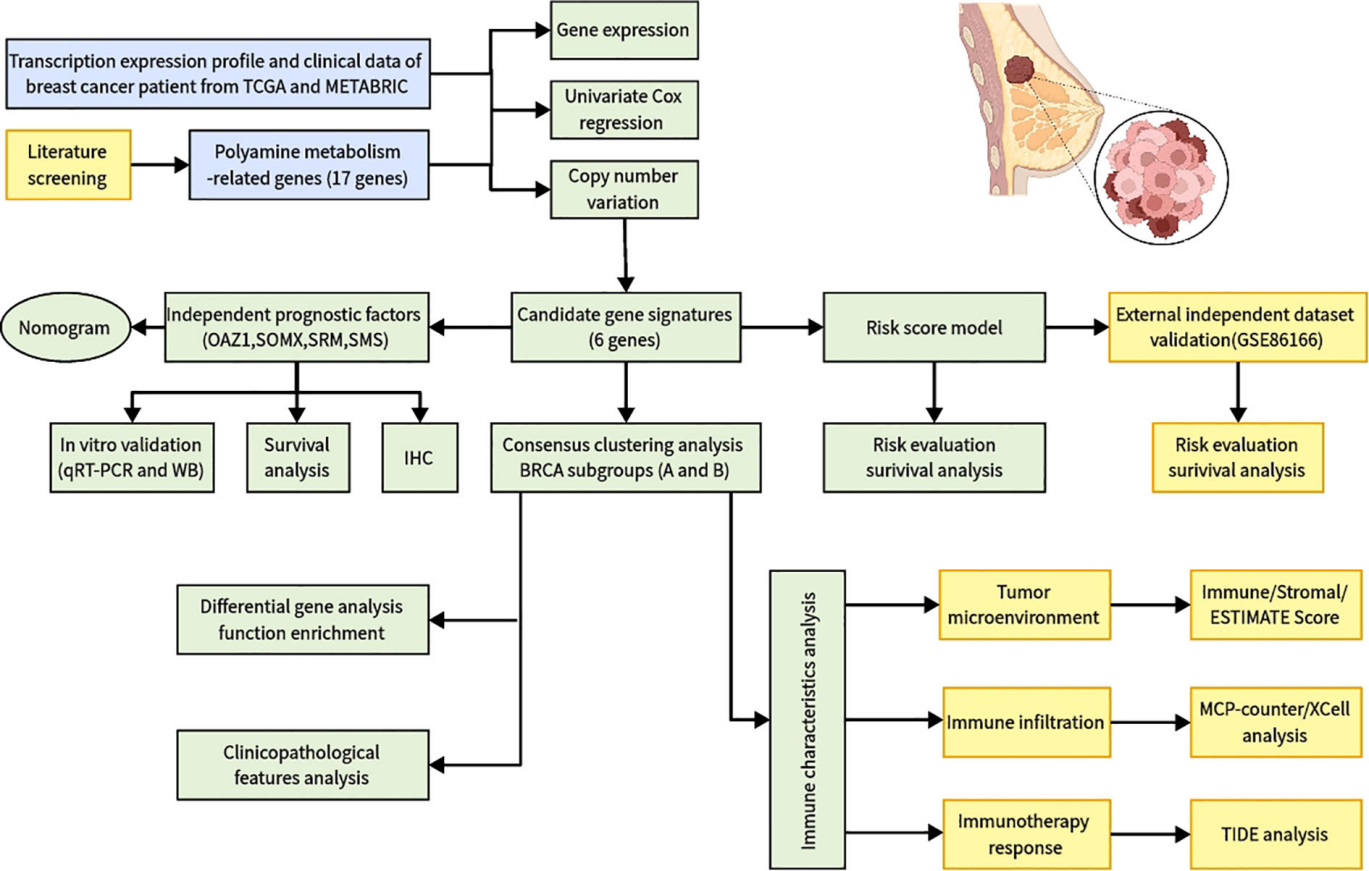
Flowchart of identifying a polyamine-related signature and four novel prognostic biomarkers in breast cancer.

**Figure 2:**
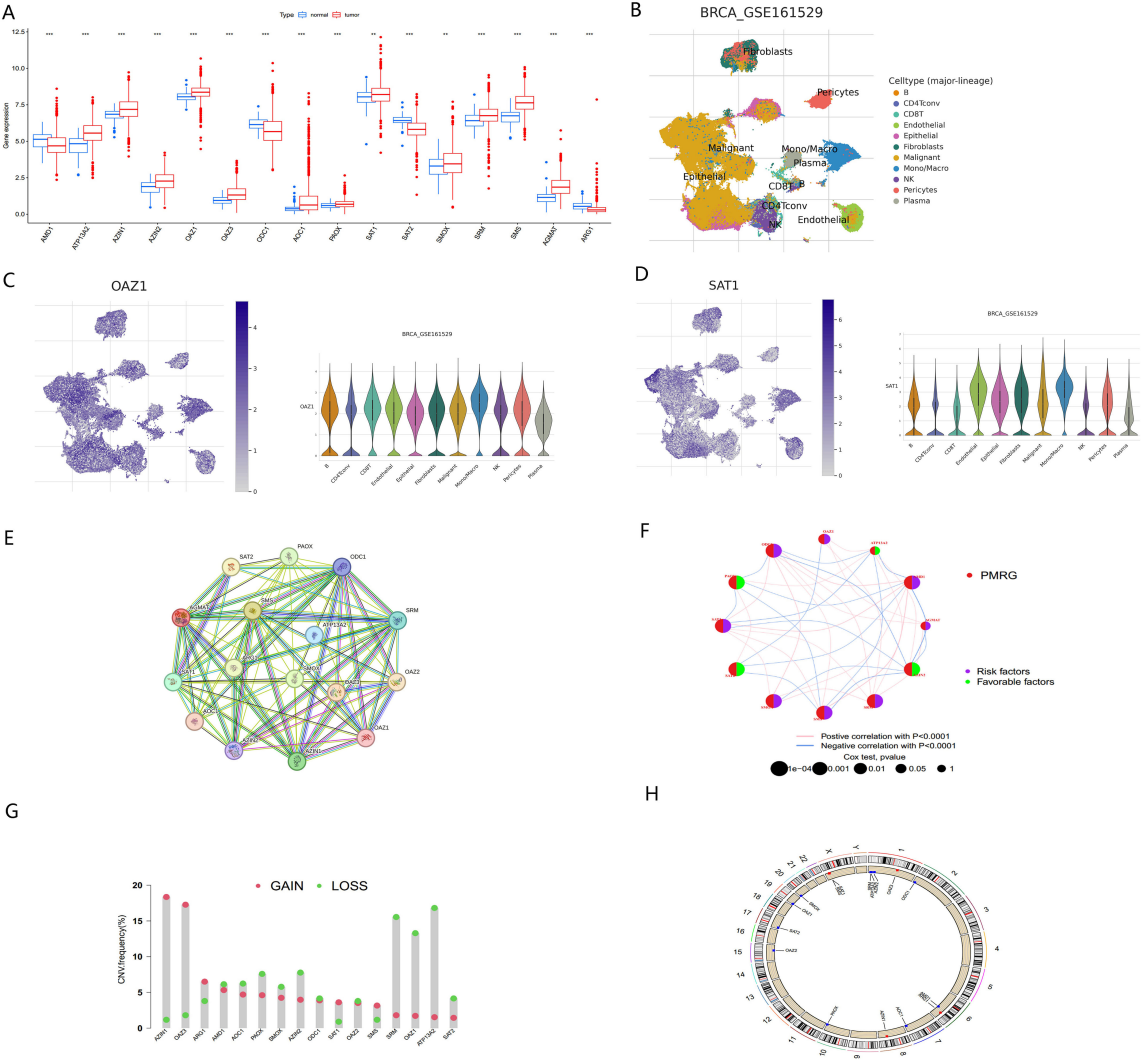
Multi-omics analysis of PMRGs in breast cancer **(A)** Expression levels of PMRGs in the TCGA-BRCA cohort. **(B)** UMAP plot of single cells in GSE161529 data set. **(C, D)** Distribution and expression patterns of OAZ1 and SAT1. **(E)** PPI network map showed the interaction of the 17 polyamine regulators. **(F)** Interaction of the polyamine regulators. Size of each cell represents the survival effect of each gene. Red represents a positive correlation, whereas blue indicates a negative correlation. **(G)** Copy number variations (CNVs) and of 17 PMRGs in TCGA-BRCA. **(H)** Chromosome region and alteration of PMRGs. **, p < 0.01; ***, p < 0.001.

The study was conducted according to the summary flowchart (**Figure 1**). A total of 17 polyamine metabolism-related genes (PMRGs) were identified from the literature. In the TCGA-BRCA cohort, 16 of these genes exhibited statistically significant differences in expression. Most polyamine metabolism genes (*ATP13A2*, *AZIN1*, *AZIN2*, *OAZ1*, *OAZ2*, *OAZ3*, *AOC1*, *PAOX*, *SAT1*, *SMOX*, *SRM*, *SMS*, and *AGMAT*) were highly expressed in BC In contrast, *AMD1*, *ODC1*, *SAT2*, and *ARG1* showed lower expression in cancer tissues (**Figure 2A**).

We further analyzed the expression levels of PMRGs at the single-cell level using the GSE161529 dataset. After quality control, 32,168 cells were annotated, including B cells, CD4Tconv, CD8T, endothelial, epithelial, fibroblasts, monocytes/macrophages (Mono/Macro), NK cells, pericytes, and plasma cells (**Figure 2B**). PMRGs were expressed explicitly in BC cells. In PMRGs, *OAZ1* and *SAT1* are highly expressed across all cell types, especially in malignant cells and immune cells (**Figures 2C, D**). *SMS* and *AMD1* are mainly expressed in malignant cells, epithelial cells and immune cells (CD8T, Mono/Macro). *SRM* is mainly expressed on epithelial cells, endothelial cells and fibroblasts, and is also expressed to a certain extent in immune cells. However, *ARG1* and *AOC1* are almost undetectable in the TME. These findings indicate that PMRGs play an important roles in BC and are closely linked to immunity (**Supplementary Figures S1, S2**).

To explore potential interactions among PMRGs, we performed a correlation analysis and constructed a protein-protein interaction (PPI) network (**Figure 2E**). *ATP13A2* is a late endolysosomal transporter and is involved in polyamine transport (8), but it did not directly interact with other proteins in our PPI network. *ATP13A2* islinked to genes in the related network, indicating that intermediate proteins maybe involved in tumorigenesis. This result is consistent with that of Tang in oral squamous cell carcinoma (29). The regulatory network provided a comprehensive view of the interconnections among the 17 PMRGs and their prognostic significance in BC. The network diagram (**Figure 2F**) illustrated the relationships among 12 survival-related genes, comprising 8 risk factors and 4 favorable factors. *AZIN2* exhibited the highest number of negative correlations (blue lines), while *ODC1* and *SRM* displayed the most positive correlations (pink lines), indicating their central regulatory roles within the PMRG network.

Copy number variation (CNV) analysis revealed frequent alterations of PMRGs in BC patients. Amplifications were most significant for *OAZ3* and *AZIN1*, located on chromosomes 1 and 8, respectively. In contrast, *ATP13A2* and *SRM* exhibited the most extensive copy number deletions, both located on chromosome 1 (**Figures 2G, H**).

### 3.2 12 polyamine metabolism-related genes were used for consistent clustering of breast cancer molecular subsets

**Figure 3:**
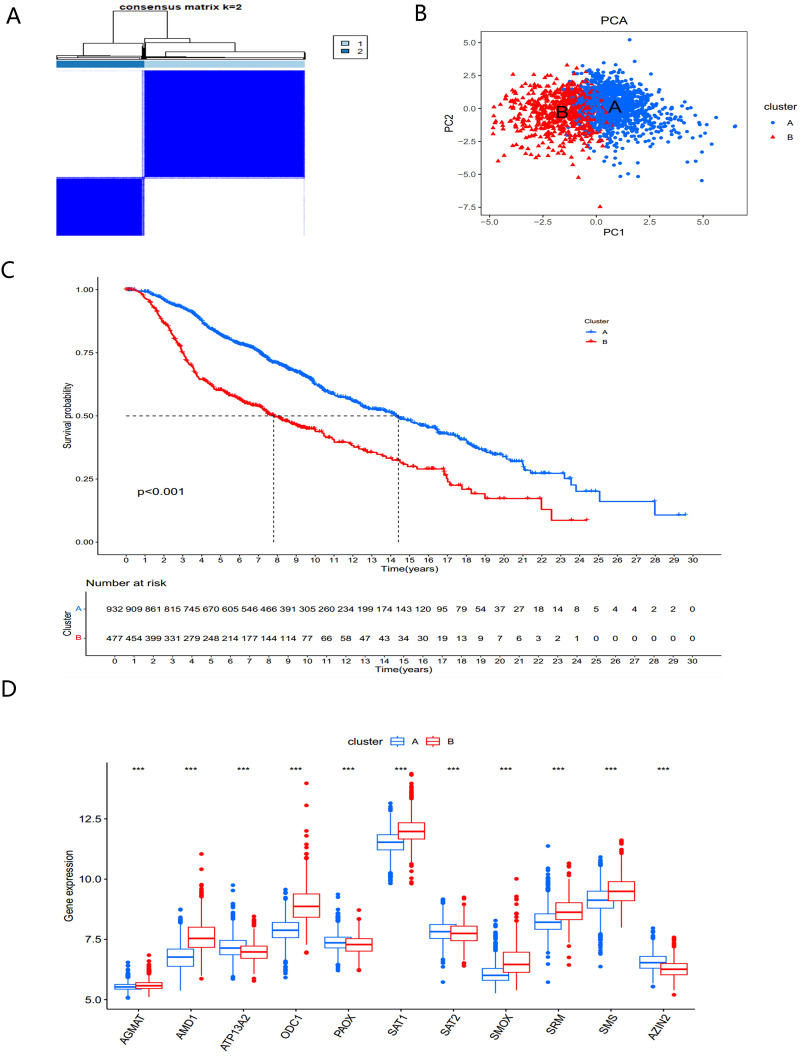
Identification and biological characteristics of PMRG expression cluster. **(A)** Consensus matrix for k = 2 was obtained by applying consensus clustering. **(B)** PCA **(C)** Kaplan–Meier curves of survival differences between the two PMRGs expression clusters. **(D)** PMRGs expression in two subtype clusters. ***, p < 0.001.

To better understand the role of PMRGs in BC we performed consensus clustering on 12 prognostic PMRGs using the “ConsensusClusterPlus” R package. As shown in **Figure 3A**, when *k*=2, the cohort was effectively divided into two distinct subtypes. Principal component analysis (PCA) revealed significant differences between the two subtypes (**Figure 3B**).

Overall survival analysis revealed significant prognostic differences between the two subtypes (*P*<0.001) (**Figure 3C**). The PMRGs-A subtype demonstrated a significant survival advantage, while PMRGs-B exhibited poorer prognosis. Expression patterns of PMRGs in the two subgroups were visualized using boxplots (**Figure 3D**), showing that most PMRGs, such as *SMS*, *SMOX*, and *SAT1*, were highly expressed in PMRGs-B. Only *ATP13A2*, *PAOX*, and *SAT2* displayed low expression, suggesting their potential as therapeutic targets.

### 3.3 Immune cell infiltration and pathways of two PMRG subtype clusters

**Figure 4:**
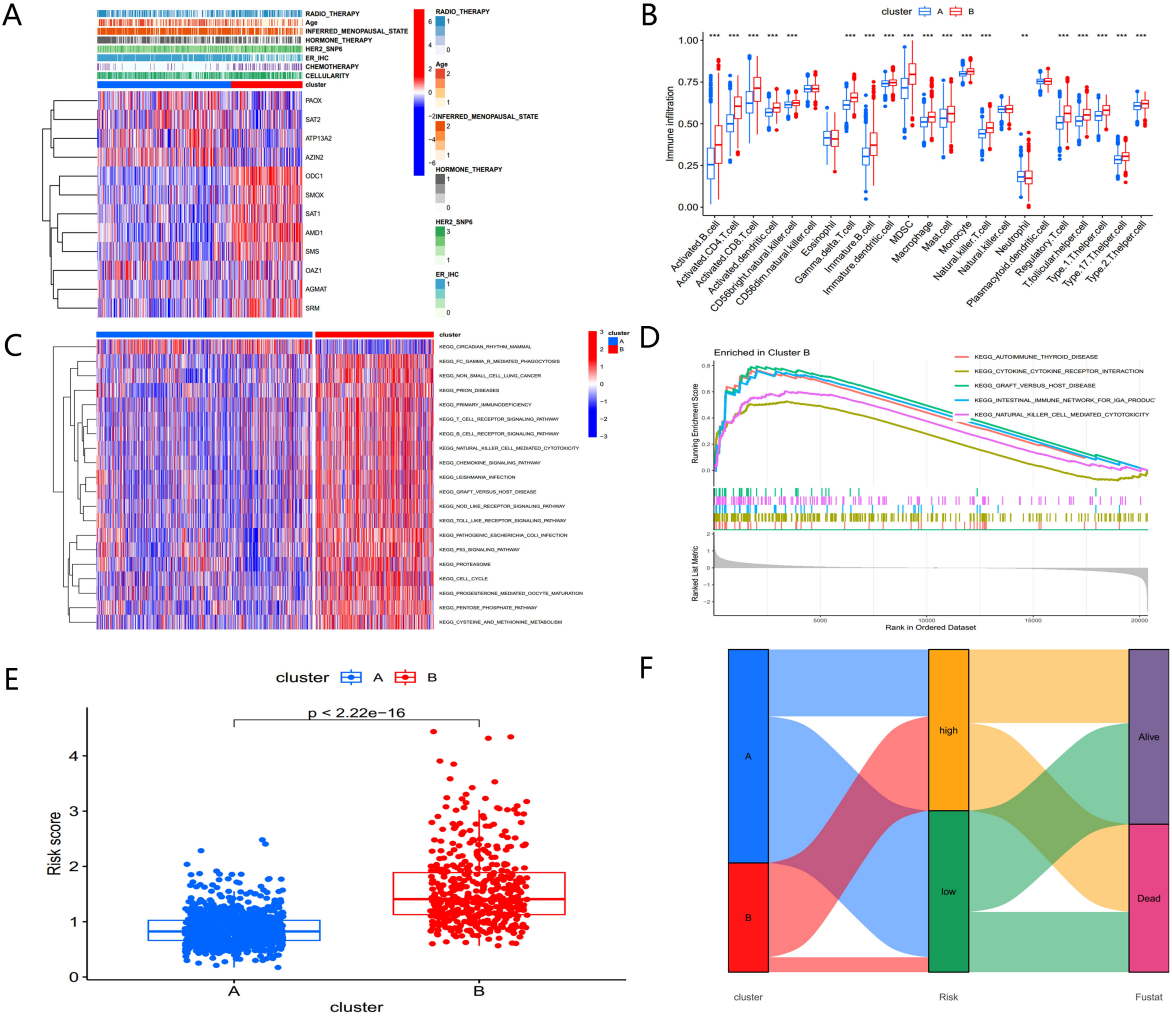
Biological characteristics of PMRG expression cluster. **(A)** Heatmap of PMRGs expression and corresponding clinicopathological features. **(B)** Immune infiltration patterns in two subtype clusters. **(C)** GSVA analysis focused on the differential enrichment of KEGG pathways between the two clusters. **(D)** GSEA enrichment analysis of PMRGs-B. **(E)** Risk scores of the two clusters. **(F)** Alluvial map of polyamine metabolic subtypes and living conditions.

A heatmap of PMRG expression and corresponding clinicopathological features for the two subtypes is shown in **Figure 4A**. To investigate immune cell infiltration differences between the subtypes, we used ssGSEA to visualize and compare 23 immune-infiltrating cell subtypes (**Figure 4B**). Significant differences were observed in immune cell infiltration between the two groups. In the high-risk PMRGs-B subgroup, there was a marked increase in immune cell infiltration, including activated B cells, activated CD4+ T cells, activated CD8+ T cells, activated dendritic cells, regulatory T cells, follicular helper cells, and type 1/17/2 helper T cells.

To explore the functional differences between the clusters, GSVA was applied to analyze KEGG pathway enrichment (**Figure 4C**). The PMRGs-B cluster, which exhibited poor prognosis, was enriched in pathways associated with the cell cycle, P53 signaling, and the pentose phosphate pathway— all of which are closely related to tumor development. GSEA enrichment analysis further confirmed that the high-risk PMRGs-B subtype was primarily enriched in autoimmune thyroid disease pathway (**Figure 4D**). There were significant differences in risk scores between the two PMRGs subtypes (**Figure 4E**), and alluvial maps (**Figure 4F**) showed changes in polyamine metabolism-related subtypes, risk scores, and life status.

These findings indicate that the two polyamine modification patterns display distinct immune infiltration characteristics and functional enrichment profiles, with PMRGs-B linked to poor prognosis and immune dysregulation.

### 3.4 Construction and validation of a well-performing prognostic signal related to polyamine metabolism

**Figure 5:**
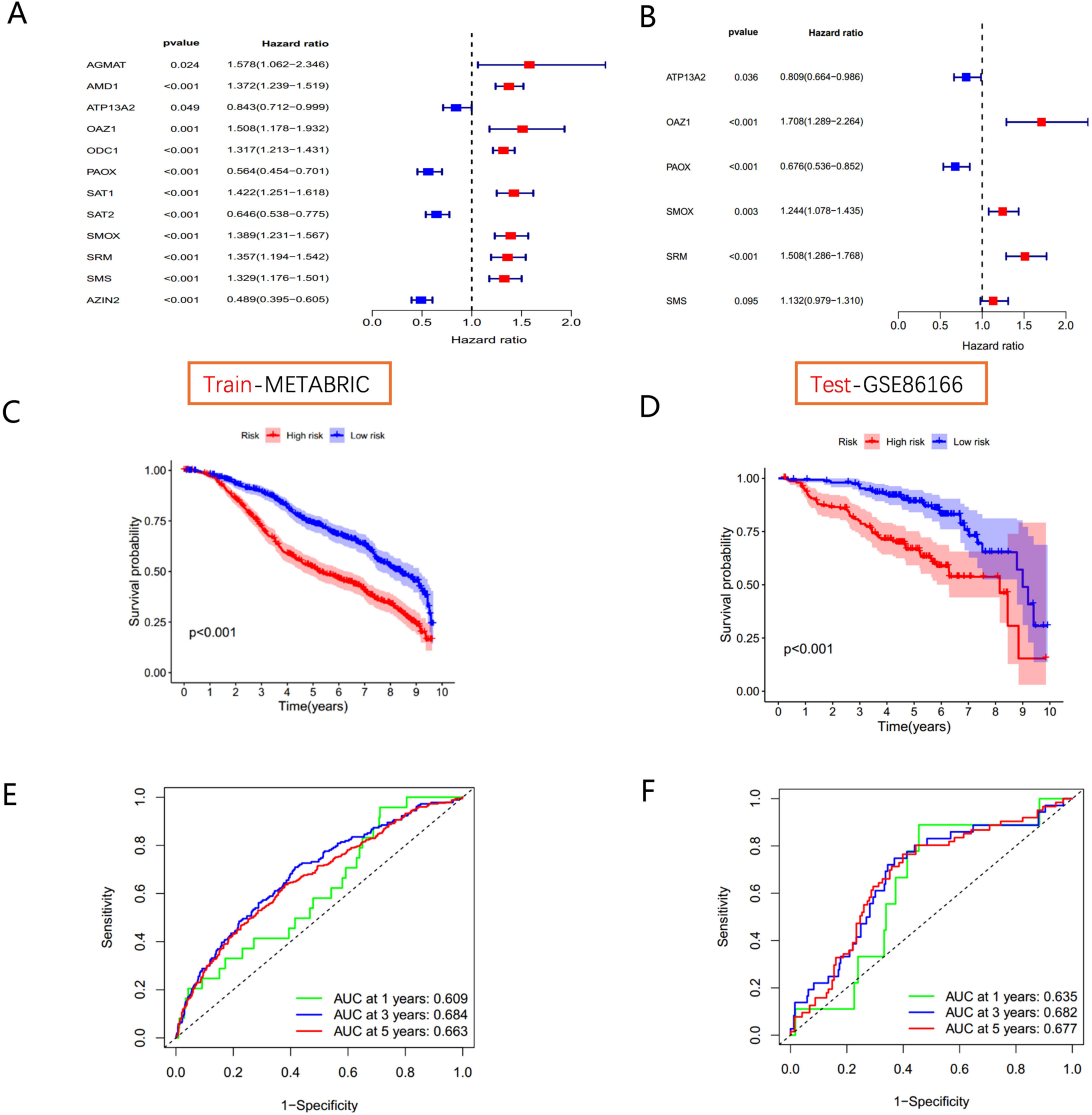
Polyamine-related risk signature construction and validation **(A)** The forest plot shows 12 PMRGs via the univariate Cox regression analysis. **(B)** The multivariate Cox regression analyses of polyamines metabolism-associated gene signatures for exploring the independent prognostic factors in BC. HR more than 1 indicates the risky gene, and HR less than 1 indicates the protective gene. **(C, D)** K-M curve of survival difference and predictive accuracy of PMRGs in the training group. **(E, F)** K–M curve of survival difference and predictive accuracy of PMRGs in the testing group.

Univariate Cox regression analysis in the METABRIC cohort (**Figure 5A**) identified 12 PMRGs significantly correlated with survival (*P*<0.05). Of these, *AGMAT*, *AMD1*, *ATP13A2*, *OAZ1*, *ODC1*, *SAT1*, *SMOX*, *SRM*, and *SMS* were risk factors (HR > 1, *P*<0.05), whereas *AZIN2*, *ATP13A2*, *PAOX*, and *SAT2* were favorable factors (HR < 1, *P*<0.05) for patient prognosis. To assess the clinical relevance of polyamine metabolism-related genes (PMRGs), we developed and validated a prognostic model using the independent cohort GSE86166. A multivariate Cox regression analysis was conducted to evaluate the effectiveness of the risk score model in predicting BC patient prognosis. Based on the median risk value, patients were divided into high- and low-risk groups. The final model consisted of six PMRGs, of which *OAZ1*, *SMOX*, *SRM*, and *SMS* were significantly correlated with overall survival (OS) and identified as risk factors (HR > 1, *P*<0.05) (**Figure 5B**).

Kaplan-Meier survival curves demonstrated that high-risk groups had significantly poorer outcomes in both the METABRIC training cohort and the GSE86166 validation cohort (**Figures 5C, D**). Time-dependent ROC curve analysis for OS at 1, 3, and 5 years showed strong predictive performance for the model (**Figures 5E, F**).

**Figure 6:**
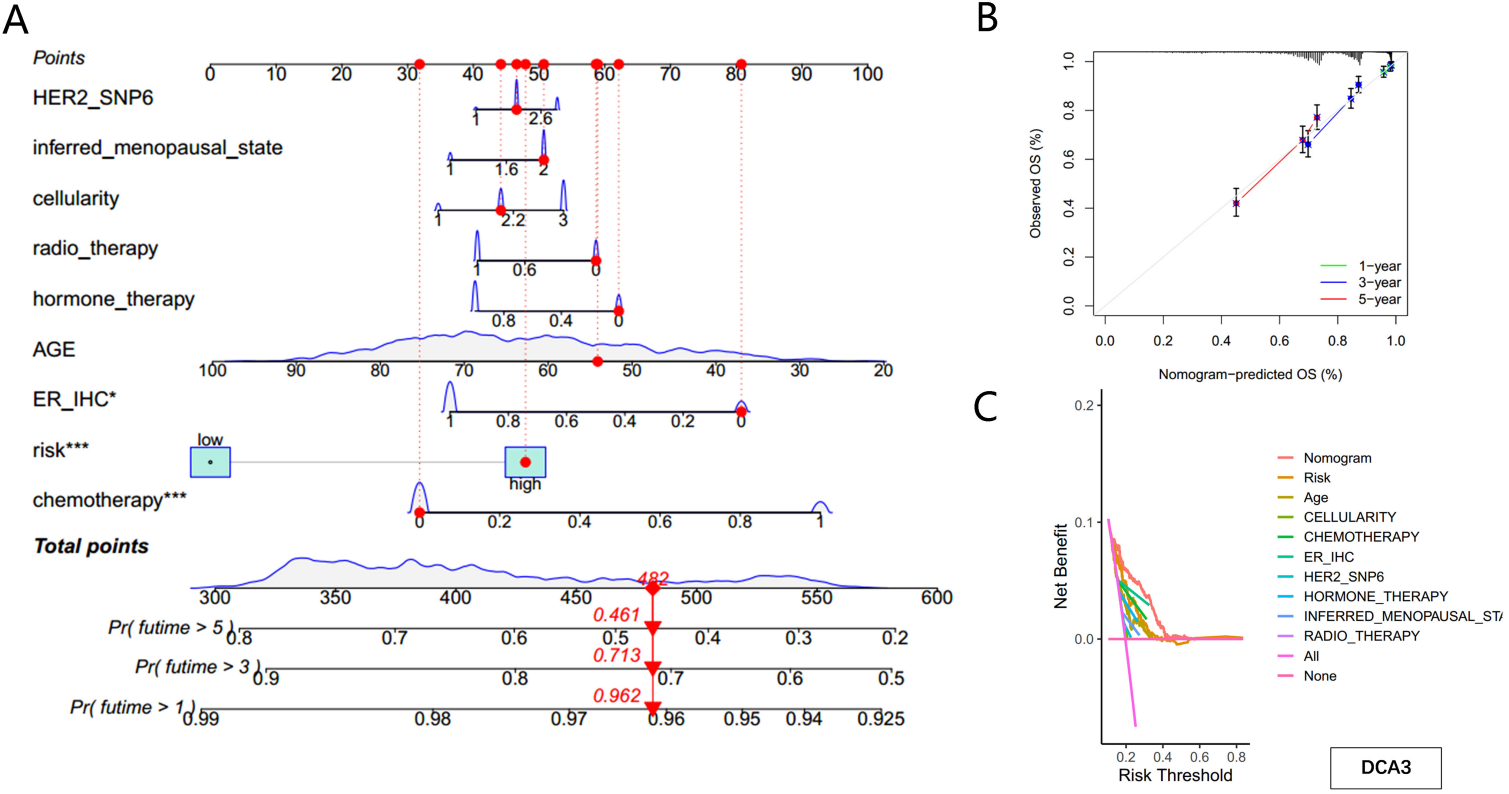
Construction of the nomogram predicting patients’ survival in BC. **(A)** Nomogram plot based on PMRG score and clinicopathological factors. **(B)** Calibration plot for the validation of the nomogram. **(C)** DCA curves of the nomogram for three-year OS in BC patients.

To account for clinicopathological factors, the PMRG-based risk score was integrated with clinical data to construct a nomogram (**Figure 6A**). The calibration plot confirmed the nomogram’s predictive accuracy (**Figure 6B**). DCA further demonstrated that the nomogram provides substantial benefits for predicting short- and long-term survival in BC patients (**Figure 6C**). These results highlight that the PMRG-based nomogram is a reliable and effective tool for predicting BC patient prognosis.

### 3.5 Identification of independent prognostic factors

Using univariate and multivariate Cox regression analyses, *OAZ1*, *SMOX*, *SRM*, and *SMS* were identified as independent prognostic factors. We further investigated the expression levels of these four genes and their diagnostic and prognostic relevance in BC tissues.

**Figure 7:**
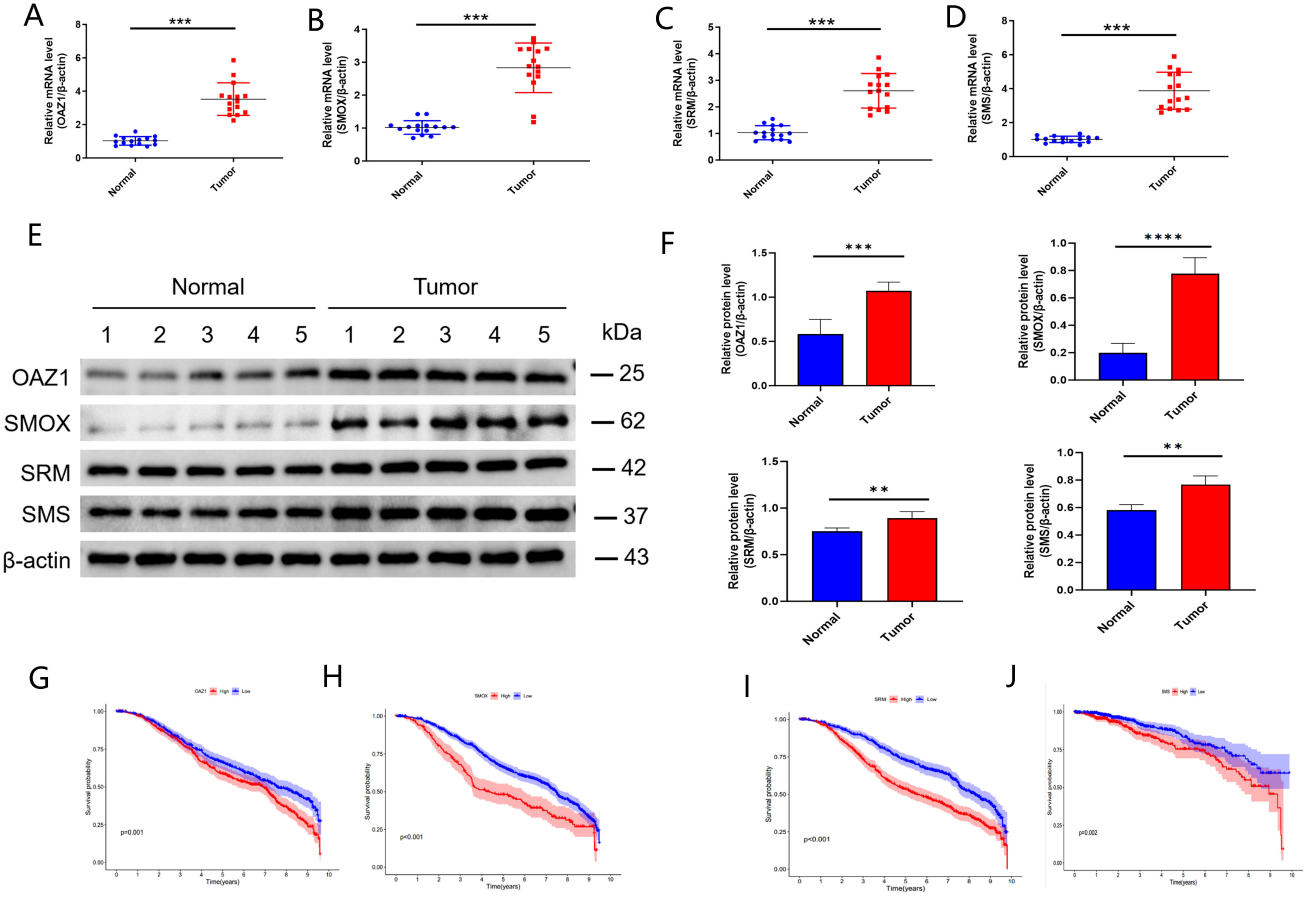
Validation of independent prognostic genes (*OAZ1*, *SMOX*, *SRM*, and *SMS*). **(A–D)** qRT‐PCR assay. **(E, F)** Western blot assay. **(G–J)** Kaplan-Meier plot depicting the predictive role of the independent genes expression for patients’ survival. **, p < 0.01; ***, p < 0.001; ****, p < 0.0001.

**Figure 8:**
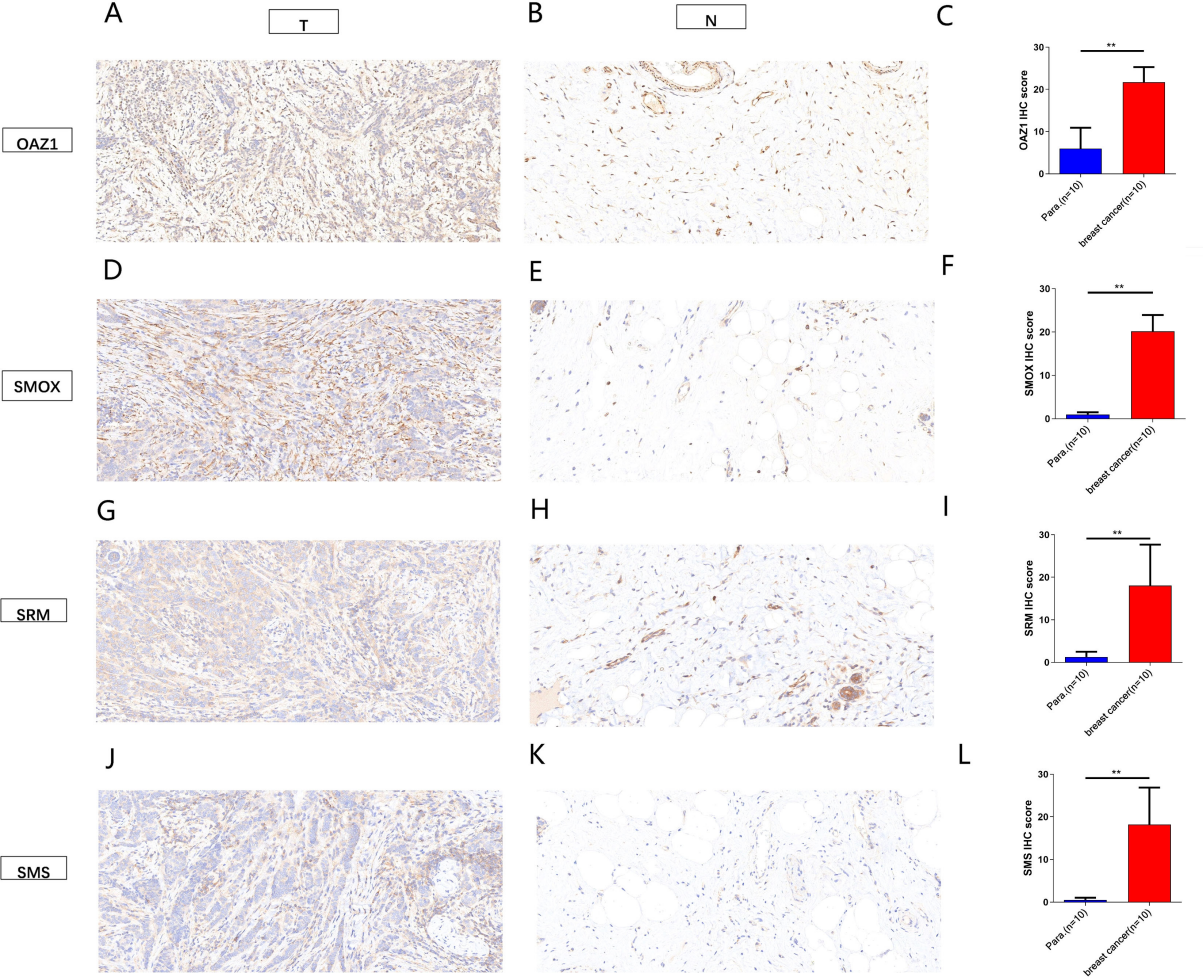
Immunohistochemical analysis showed the expression of four independent factors in para-carcinoma tissues and breast cancer tissue (magnification 200x). **(A–C)** *OAZ1*. **(D–F)** *SMOX*. **(G–H)** *SRM*. **(J–L)** *SMS*. **, p < 0.01.

The expression levels of *OAZ1*, *SMOX*, *SRM*, and *SMS* were significantly elevated in cancer tissues compared to normal tissues (**Supplementary Figures S3A–D**), as confirmed by qRT-PCR (**Figures 7A–D**), Western blot (**Figures 7E, F**), and immunohistochemistry (**Figure 8**). Survival analysis revealed that patients with high expression of these four genes had a significantly poorer prognosis (**Figures 7G–J**).

These findings establish *OAZ1*, *SMOX*, *SRM*, and *SMS* as potential biomarkers and independent prognostic factors in BC providing critical insights for future clinical applications.

### 3.6 Gene set enrichment analysis and immune activity of different risk scores

**Figure 9:**
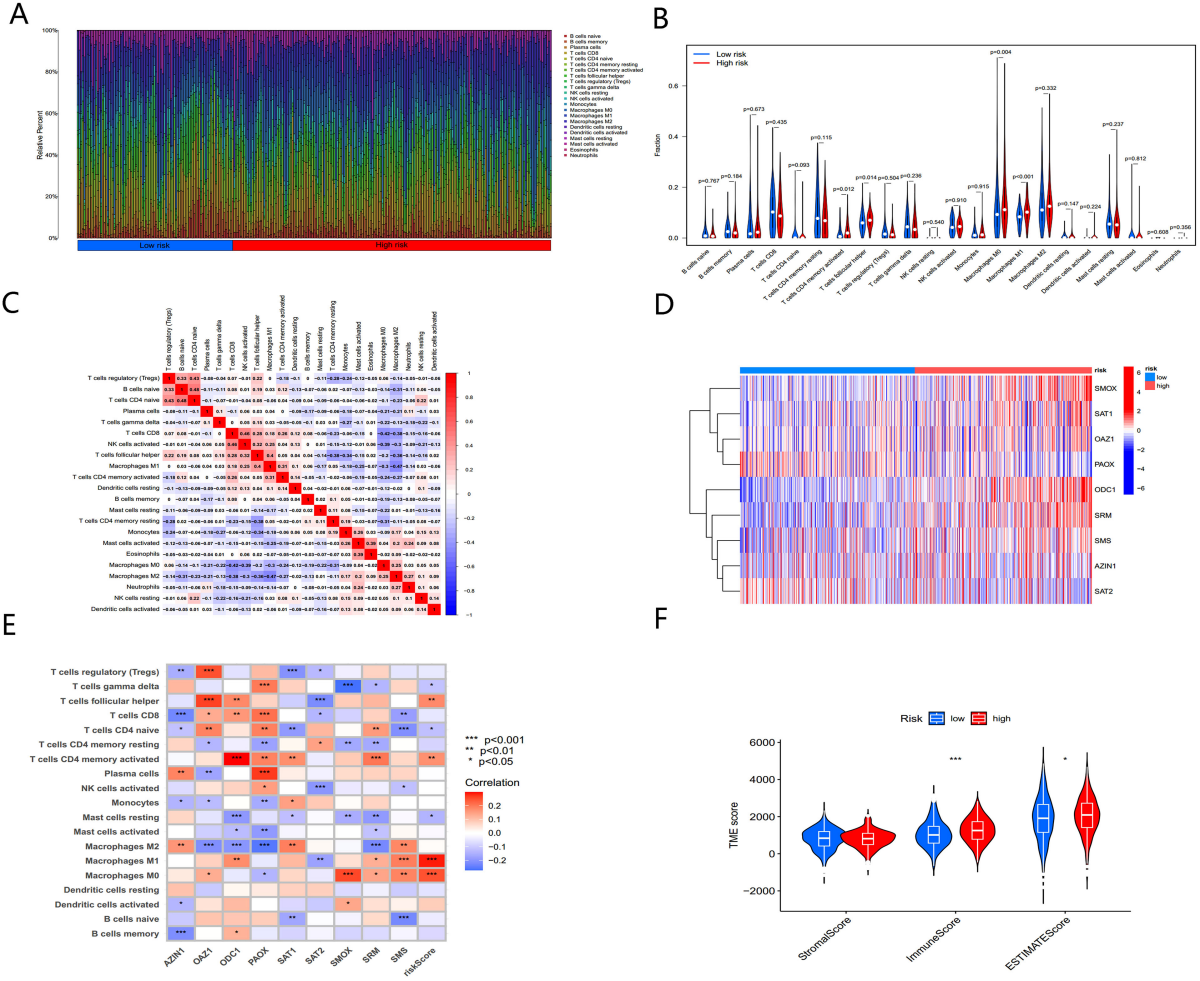
The immune microenvironment of BC tissues at different risk scores. **(A)** The relative proportion of infiltrating immune cells with different risk score. **(B)** Immune cell component between high-risk group and low-risk group. **(C)** Correlation between immune cells. **(D)** Heatmap showing the expression patterns of nine hub PMRGs. **(E)** Correlation between immune cells and nine hub PMRGs. **(F)** Estimate score of the expression profile in high-risk group and low risk group. *, p < 0.05; ***, p < 0.001.

The immune microenvironment plays a crucial role in tumor progression and response to immunotherapy. We used ssGSEA to analyze the expression of 22 immune-infiltrating cell types in association with risk scores. BC samples were ranked from low to high-risk scores, showing the proportion of different immune cells across the risk spectrum (**Figure 9A**). Macrophage M0 composition accounted for the largest proportion of immune cells in the high-risk group (**Figure 9B**), suggesting that macrophages M0 may contribute significantly to the poor prognosis of BC patients.

Further investigation of the regulatory relationships between immune cells (**Figure 9C**) revealed a significant negative correlation (cor = -0.42) between macrophages M0 and CD8+ T cells. The nine-gene signatures used to construct the PMRG-based model showed distinct expression patterns in high- and low-risk populations (**Figure 9D**) and were significantly associated with various immune cell infiltrations (**Figure 9E**).

We next analyzed the tumor microenvironment (TME) of BC patients in the high- and low-risk groups. By estimating expression-based scores, we obtained the stromal score, immune score, and ESTIMATE score for both groups (**Figure 9F**). The tumor mutation burden (TMB) was significantly higher in the high-risk group compared to the low-risk group, indicating that higher risk scores correlate with higher TMB.

**Figure 10:**
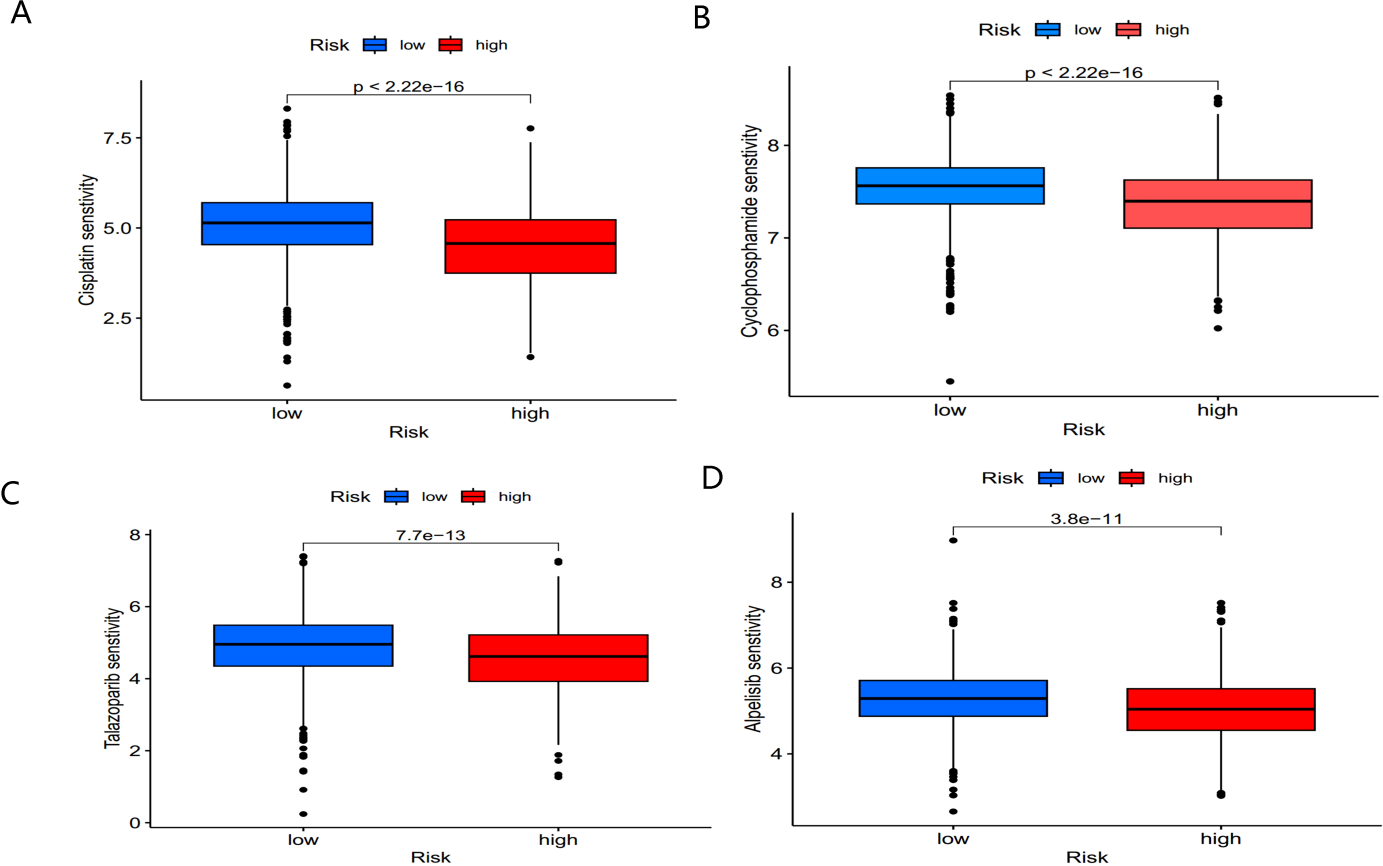
Sensitivity of low- and high-risk patients to four common chemotherapy agents. **(A)** Cisplatin, **(B)** Cyclophosphamide, **(C)** Talazoparib, and **(D)** Alpelisib.

Chemotherapy remains a cornerstone of cancer treatment. Using the “oncoPredict” R package, we explored potential differences in chemotherapy sensitivity between the high- and low-risk groups. High-risk patients exhibited greater sensitivity to common chemotherapy drugs, such as Cisplatin and Cyclophosphamide (**Figures 10A, B**). Additionally, small molecule drugs targeting PARP inhibitors (e.g., Talazoparib) and PI3K inhibitors (e.g., Alpelisib) were found to be more effective in high-risk patients (**Figures 10C, D**).

These findings suggest that BC patients in the high-risk group not only exhibit distinct immune microenvironment characteristics but also show promising sensitivity to chemotherapy and targeted therapies, offering potential clinical benefits for treatment optimization.

## 4 Discussion

Breast cancer is a heterogeneous and highly aggressive disease, ranking among the leading causes of global morbidity and mortality (11) and representing a significant disease burden for women worldwide (37). Polyamines (PAs) play a critical role in the proliferation of breast cancer cells (4). Although intracellular PA concentrations are tightly regulated, dysregulation frequently occurs in breast cancer cells, making polyamine metabolism (PM) a potential target for BC intervention. PM is closely associated with the tumor microenvironment (TME) and is involved in anti-tumor immunity (38, 39), with intricate crosstalk between PM and the TME. However, the role of PM in the BC TME remains underexplored (23). Therefore, this study investigated the clinical significance of polyamine metabolism-related genes (PMRGs) in BC.

In this study, we analyzed PMRG expressions in BC at transcriptome and single-cell levels. Most PMRGs were overexpressed in BC samples, and a majority (12/17) were associated with survival. Given the heterogeneity of BC, patients were stratified based on the expression of 17 PMRGs using consensus clustering. Two distinct PMRG expression subtypes were identified, showing significant differences in prognosis and biological pathways. Patients with high-risk scores have the worse OS related to poor survival prognosis and high TMB than those with low risk scores and inhibitory TME status. These reveal that the risk score can offer an innovative approach to assessing the TME status and prognosis of breast cancer.

Our analysis revealed that the PMRG expression cluster B subtype had a poorer prognosis and was significantly enriched in tumor- and immune-related pathways. These included the cell cycle, natural killer cell-mediated cytotoxicity, T cell receptor signaling pathway, chemokine signaling pathway, P53 signaling pathway, and the pentose phosphate pathway (PPP). The P53 signaling pathway plays a pivotal role in regulating breast cancer progression. For example, activation of P53 by Salt-inducible kinase 1 (SIK1) promotes oxidative phosphorylation, thereby inhibiting aerobic glycolysis and suppressing cell proliferation in breast cancer (40). Conversely, inhibition of the P53 signaling pathway contributes to breast cancer progression by increasing cell proliferation, migration, and invasion (41).The pentose phosphate pathway (PPP) also plays an essential role in breast cancer metabolism, contributing to oxidative stress regulation, nucleotide synthesis, and maintenance of the REDOX state (41, 42). It has been implicated in various cancer cell processes, including proliferation, apoptosis, drug resistance, invasiveness, metastasis, and senescence (43–47). By maintaining a high proliferative state, the PPP supports cancer cell viability (48, 49).

We developed a risk score model established by six genes (*OAZ1*, *SMOX*, *SRM*, *SMS*, *ATP13A2*, and *PAOX*) from the METABRIC and GEO database via multivariate COX correlation analysis, predicting the different prognosis of the high- and low- risk patients. Survival analysis indicates that this risk model can effectively distinguish high-risk and low-risk patients. To improve the accuracy of model prediction, this study combined prognostic risk scores with clinical characteristics to construct the nomogram for OS prediction. Through analysis of ROC curves and DCA, we found that Nomogram had a better predictive ability to predict BC prognosis than other indicators. We observed a significant positive correlation between risk scores and chemotherapy, as well as ER_IHC. This finding enhances the prediction accuracy and clinical significance of the risk model.

The occurrence and metastasis of tumors are closely related to the tumor microenvironment (TME) (50). In breast cancer TME, tumor-associated macrophages (TAMs) play a crucial role, constituting more than 50% of the tumor volume (51). TAMs are typically activated within tumors, where they exert tumor-promoting (52, 53) and immunosuppressive effects (54), ultimately leading to poor prognosis and chemotherapy resistance (55, 56).

Given the established relationship between polyamines and immunity, we analyzed immune cell infiltration in high- and low-risk groups using ssGSEA, CIBERSORT, and ESTIMATE algorithms. Immune cell infiltration was elevated in the high-risk group, particularly in macrophages M0, M1, T cells CD4 memory activated, and T cells follicular helper (Tfh) cells. Monocytes differentiate into three macrophage subtypes: non-activated M0 macrophages, pro-inflammatory M1 macrophages, and anti-inflammatory M2 macrophages. M1 macrophages release potent pro-inflammatory cytokines, such as TNF-α, IL-1, IL-6, IL-12, and iNOS, contributing to chronic inflammation and fibrous capsule formation. In contrast, M2 macrophages promote anti-inflammatory responses, tissue repair, and growth through the release of factors like IL-4, IL-10, and TGF-β (57–59).

Numerous previous studies have shown that M2 macrophages promote proliferation of breast cancer cells (54), renal tubular cells (60), colon cancer cells (61). Tfh cells provide essential help to B cells for effective antibody-mediated immune responses. In various solid organ tumor types of non-lymphocytic origin, their presence frequently coincides with a better prognosis. Existing studies confirm that Tfh cells is a key to the success of the immune checkpoint blockade (ICB) determinants and predictors (62).

Additionally, the high-risk group demonstrated a relatively favorable immunotherapy response based on the TIDE score. These results suggest that PMRG signatures may serve as predictors of immunotherapy responses. Given the dependency of tumor cells on polyamines and the critical physiological roles of polyamines in various immune cell types, targeting polyamine metabolic pathways may enhance immunotherapy efficacy (23, 24, 29, 63).

This study first developed and validated the important biological function of PMRG signatures in determining the prognosis of BC patients. More importantly, candidate genes *OAZ1*, *SMOX*, *SRM*, and *SMS* were identified as independent prognostic factors. We further validated their expression levels in BC cells and explored their prognostic value using survival analysis.

*OAZ1* is a key member of the ornithine decarboxylase enzyme family involved in polyamine metabolism (64). *OAZ1* exhibits tumor inhibitory activity by affecting cell proliferation, apoptosis, and differentiation in oral cancer cell lines, leukemia, and non-small cell lung cancer (65–67). However, its role in breast cancer remains poorly understood.

*SMOX* (Spermine Oxidase) plays a major role in the catabolism of mammalian polyamines by oxidizing spermine to spermidine, producing reactive oxygen species (ROS) in the process (68). ROS induced by oxidative stress can lead to epithelial cell apoptosis but also cause DNA damage, thereby increasing the risk of tumorigenesis (69, 70). High *SMOX* expression has been implicated in gastric cancer (71), hepatocellular carcinoma (72), and colorectal cancer (73). *SMOX* is also associated with drug responses and cellular reactions to stress stimuli.

*SRM* (Spermidine Synthase) converts putrescine to spermidine in the polyamine biosynthesis pathway. *SRM* is overexpressed in prostate cancer and clear cell renal cell carcinoma (ccRCC), serving as a reliable biomarker and therapeutic target (74, 75). Overexpression of *SRM* may increase bladder cancer resistance to pirarubicin, while *SRM* knockdown improves chemotherapy efficacy (64). *SRM* also regulates the immune microenvironment, as its knockdown inhibits fibroblast proliferation (76). Recent studies have shown that targeting *SRM* can enhance the sensitivity of FGFR-mutant bladder cancer cells to erdafitinib treatment (77).

*SMS* (Spermine Synthase) is the only enzyme responsible for spermine (SPM) synthesis in mammalian cells (15). As the final step in the polyamine biosynthesis pathway, *SMS* catalyzes the transfer of aminopropyl from decarboxylated S-adenosylmethionine (dcSAM) to spermidine (SPD) to produce spermine (78). Mutations in *SMS* cause Snyder-Robinson Syndrome (SRS), a rare X-linked recessive disorder characterized by intellectual disability, developmental delays, skeletal abnormalities, and seizures (78–80). Research on *SMS* in cancers is limited; however, existing studies indicate that *SMS* overexpression promotes colon and pancreatic cancer progression (81, 82). Targeting *SMS* with inhibitors reduces polyamine levels, thereby suppressing tumor cell proliferation (83).

In summary, this study, for the first time, developed and validated the critical biological function of polyamine metabolic gene markers in predicting BC patient prognosis. The nomogram based on this model offers a valuable tool for clinicians to develop personalized treatment plans for BC patients in clinical practice. Importantly, candidate genes *OAZ1*, *SMOX*, *SRM*, and *SMS* were identified as independent prognostic factors. Future research into the molecular mechanisms underlying these markers, along with prospective randomized clinical trials, will have significant clinical implications and provide a roadmap for precision medicine in breast cancer.

## Data Availability

The original contributions presented in the study are included in the article/**Supplementary Material**. Further inquiries can be directed to the corresponding author.

## Supplementary material

Supplementary Figure 1-2 Expression of *SAT2*, *AZIN1*, *SMS*, *OAZ2*, *SRM*, *AMD1*, *ODC1*, *ATP13A2*, *SMOX*, *AOC1*, *OAZ3*, *AGMAT*, *ARG1*, *PAOX*, *AZIN2* in single cell data set GSE161529.

Supplementary Figure 3 The expression of independent factors was upregulated in breast cancer tissues in TCGA dataset. **(A)** *OAZ1*, **(B)** *SMOX*, **(C)** *SRM*, and **(D)** *SMS*.
