# Supplementary figures and images for: Polyamine metabolism related gene index prediction of prognosis and immunotherapy response in breast cancer
Source: Front Oncol. 2025 Jul 31;15:1613458. doi: 10.3389/fonc.2025.1613458 (PMC12350266; doi:10.3389/fonc.2025.1613458)

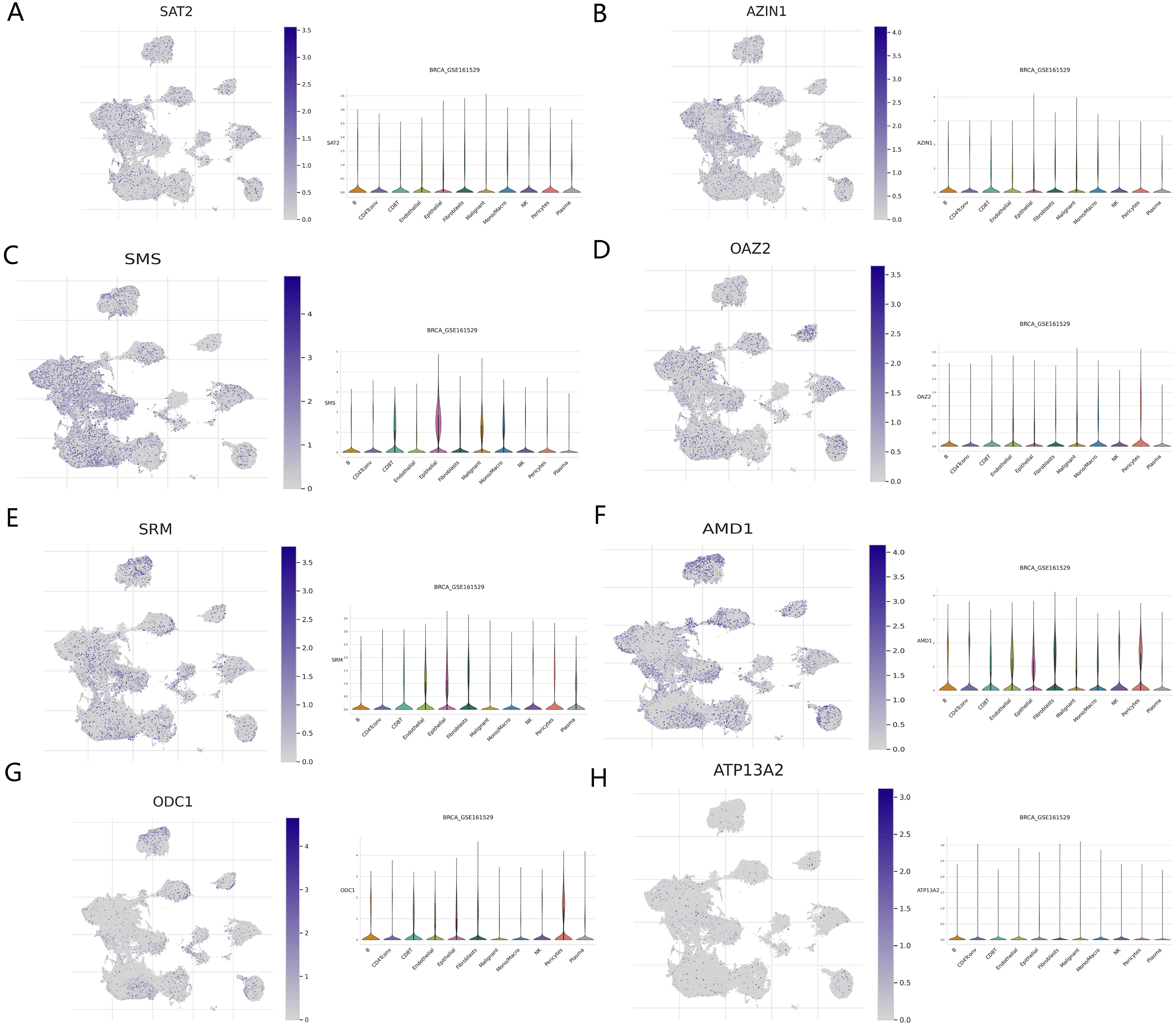

Supplement: Supplementary Figure 1-2 — Expression of SAT2, AZIN1, SMS, OAZ2, SRM, AMD1, ODC1, ATP13A2, SMOX, AOC1, OAZ3, AGMAT, ARG1, PAOX, AZIN2 in single cell data set GSE161529. [file Image1.jpeg]

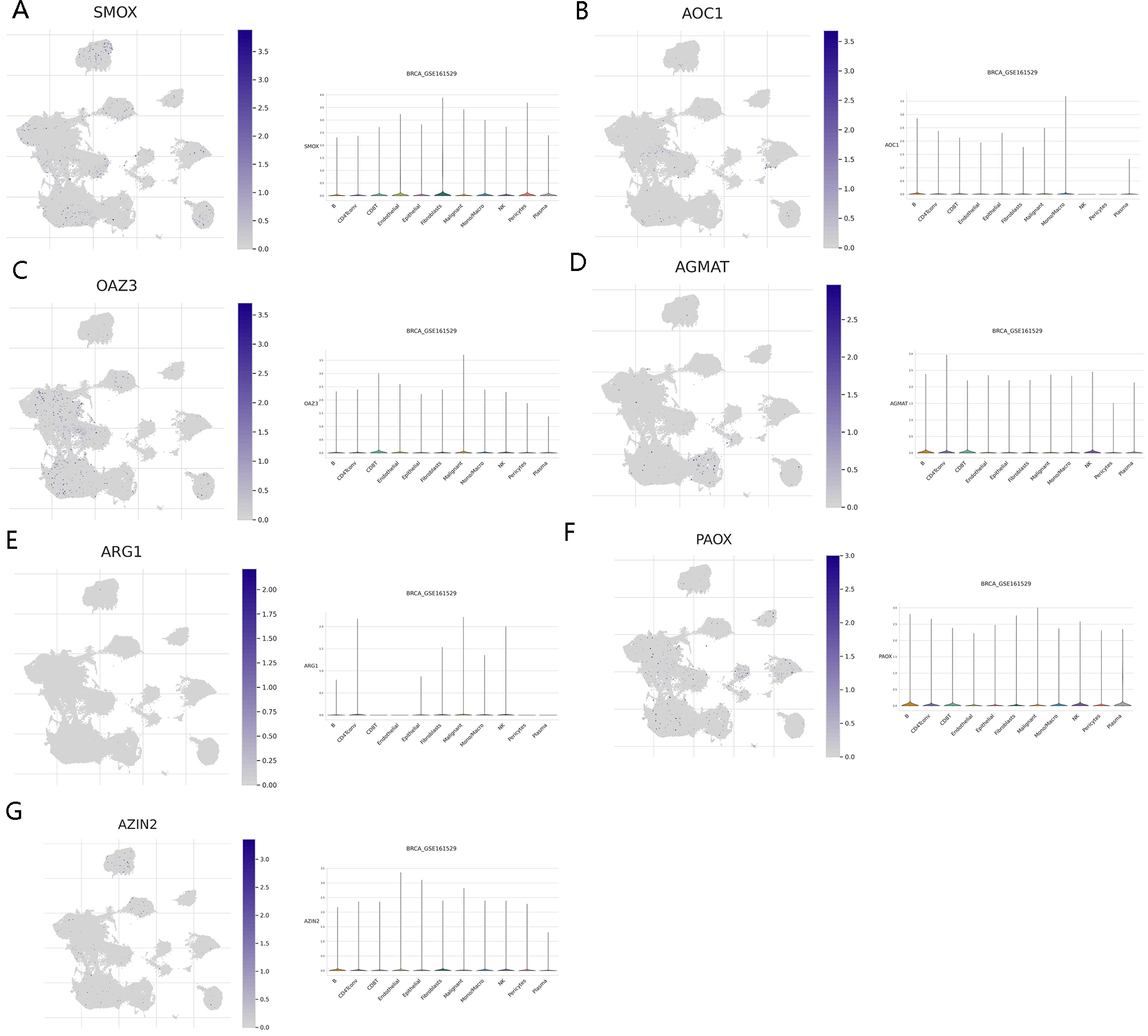

Supplement: Supplementary Figure 3 — The expression of independent factors was upregulated in breast cancer tissues in TCGA dataset. (A) OAZ1, (B) SMOX, (C) SRM, and (D) SMS. [file Image2.jpeg]

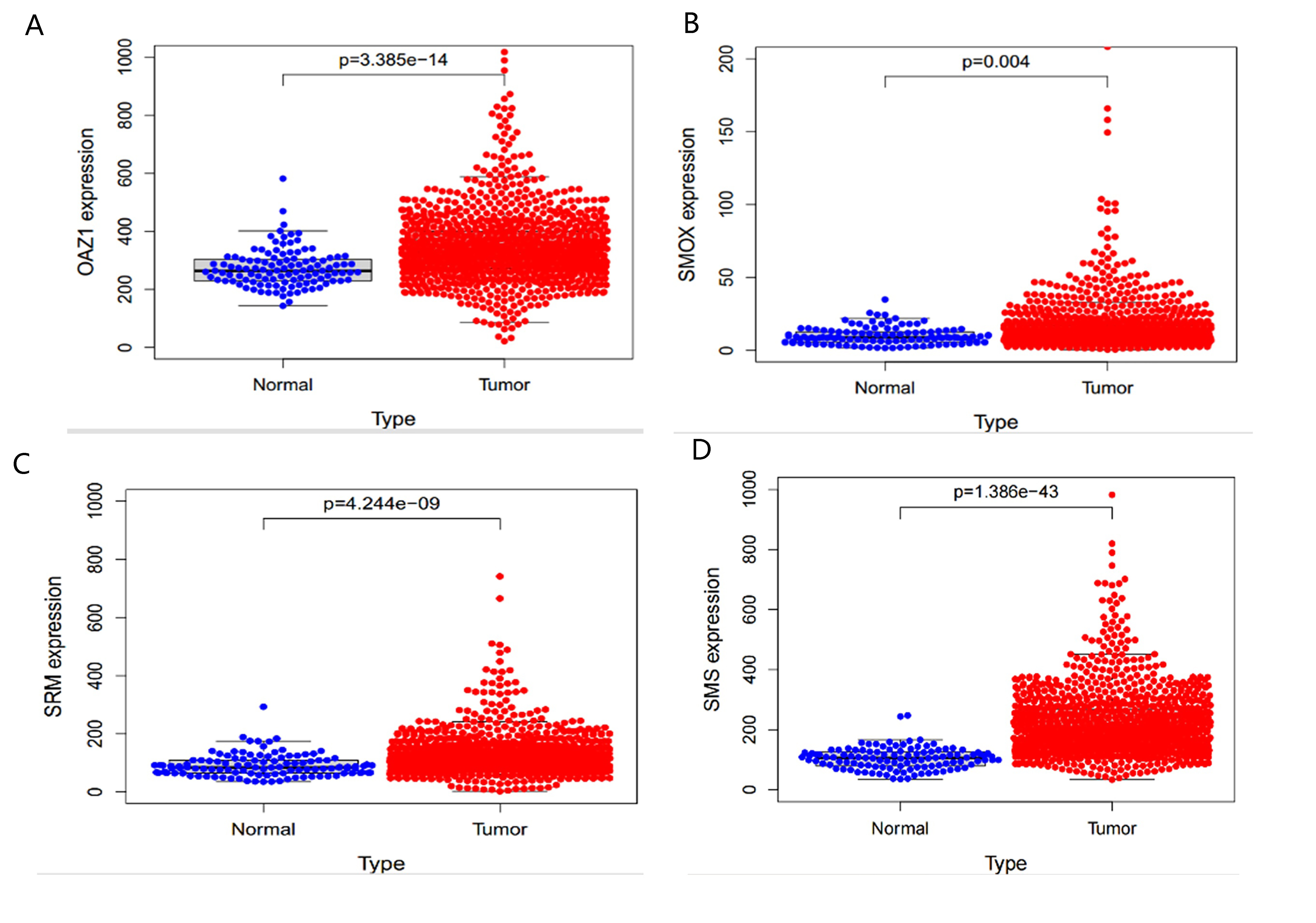

Supplement: Supplementary file 3 [file Image3.jpeg]
